# Review on the Increasing Role for PSMA-Based Radioligand Therapy in Prostate Cancer

**DOI:** 10.3390/cancers16142520

**Published:** 2024-07-12

**Authors:** Finn Edler von Eyben, Irene Virgolini, Richard Baum

**Affiliations:** 1 Center of Tobacco Control, 5230 Odense, Denmark; 2 Department of Nuclear Medicine, University Hospital Innsbruck, 6020 Innsbruck, Austria; 3 DKD Helios Clinic, 65 191 Frankfurth-Wiesbaden, Germany

**Keywords:** combination therapy, nuclear medicine, prostate neoplasm, prostate-specific membrane antigen, randomized controlled trials

## Abstract

**Simple Summary:**

In 2021, the randomized controlled trial VISION showed that prostate-specific membrane antigen (PSMA)-based third-line radioligand therapy as monotherapy for patients with metastatic castration-resistant prostate cancer with positive lesions on PSMA PET/CT increases overall survival relative to the comparator treatment. The good results have inspired new trials. Our review provides a comprehensive update on post-VISION trials on RLT. The results are promising and may expand the role of PSMA-based RLT in patients with prostate cancer.

**Abstract:**

In 2021, two randomized controlled trials (RCTs), TheraP and VISION, demonstrated that ^177^Lu-PSMA-617 as monotherapy was more effective for the decline of PSA than the comparator third-line treatments. Methods: Our review summarizes new RCTs that add to the use of radioligand therapy (RLT) for patients with high-risk prostate cancer (PCa). Results: Four past and present RCTs included 1081 patients. An RCT, ENZA-p, studied first-line treatment of patients with metastatic castration-resistant PCa (mCRPC). A combination of enzalutamide (ENZA) and ^177^Lu-PSMA-617 gave longer progression-free survival than ENZA as monotherapy. Other RCTs of patients with mCRPC, including the PSMAfore, and SPLASH trials, showed ^177^Lu-PSMA-617 as second-line treatment gave better progression-free survival than androgen receptor pathway inhibitors (combined *p* value < 6.9 × 10^−6^). Conclusions: Patients with PCa gain if they are given PSMA-RLT early in the treatment of PCa and as part of combination therapies.

## 1. Introduction

Prostate cancer (PCa) is a frequent male cancer and has the highest cancer mortality among men next to lung cancer. So, efforts to reduce the mortality are important. Pilot trials of neoadjuvant treatment with two androgen receptor pathway inhibitors (ARPIs), enzalutamide (ENZA) and darolutamide, reported positive results [1,2]. Neoadjuvant treatment can reduce the five-year risk of PSA relapse (PSAR, biochemical recurrence, BCR) after the routine initial treatments by up to 50%.

**Figure 1:**
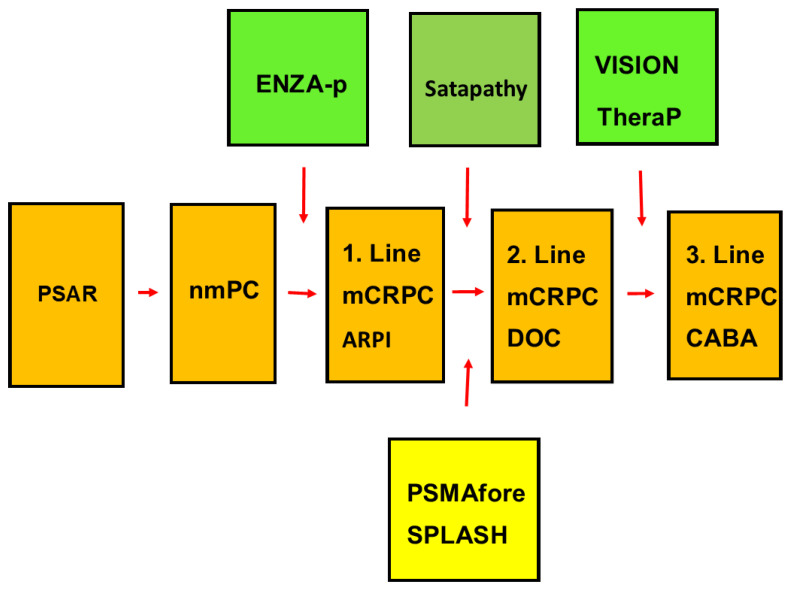
Flow scheme of phases of progressive prostate cancer from PSA relapse (PSAR), over non-metastatic PCa (nmPC), to metastatic castration-resistant PCa (mCRPC). The Figure shows the traditional sequence of phases of recurrent PCa. The most common first-line treatment of mCRPC (1. Line) is an androgen receptor pathway inhibitor (ARPI). The most common second-line treatment (2. Line) is docetaxel (DOC), and third-line treatment (3. Line) is cabazitaxel (CABA). Four randomized controlled trials (RCT) of PSMA-RLT, including VISION, TheraP, and ENZA-p, are reported in full-length publications and are shown with a green background [14,15,23]. Two other RCTs, PSMAfore and SPLASH, have been presented at major international conferences, and are shown with a yellow background.

Without neoadjuvant treatments, up to half of the patients who initially were treated with radical prostatectomy (RP) or radiation therapy (RT) had a recurrence of PCa. The first phase is PSA relapse (PSAR, biochemical recurrence, BCR), as indicated in Figure 1. The second phase is non-metastatic PCa (nmPC). For patients with nmPC, a combination of androgen deprivation therapy (ADT) and androgen receptor pathway inhibitors (ARPIs) prolongs recurrence-free survival more than ADT as the only treatment [3,4,5,6].

After ADT, the next phase is metastatic castration-resistant PCa (mCRPC). In real-world analyses, ARPIs (especially ENZA or abiraterone (ABI)) were first-line treatment for two-thirds of mCRPC patients, and docetaxel (DOC) was first-line treatment for a sixth to a third of the patients [7,8]. The CARD trial was a randomized controlled trial (RCT) that investigated the third-line treatment of mCRPC patients who had failed with docetaxel (DOC) and an ARPI [9]. Patients given cabazitaxel (CABA) lived longer than those given a second ARPI: median 13 months versus 11 months, respectively. Real-world studies of third-line treatment confirmed that CABA gives a better outcome than a second ARPI [10,11]. 

A trend for treatment is early use of drugs that are effective as third-line treatments [12]. RCTs for patients with mCRPC supported doublets of ADT and ARPI [13] and triplets of ADT, ARPI, and DOC. For patients with metastatic hormone-sensitive PCa (mHSPC), RCTs support adding DOC to the ADT and ARPI doublet. 

**Figure 2:**
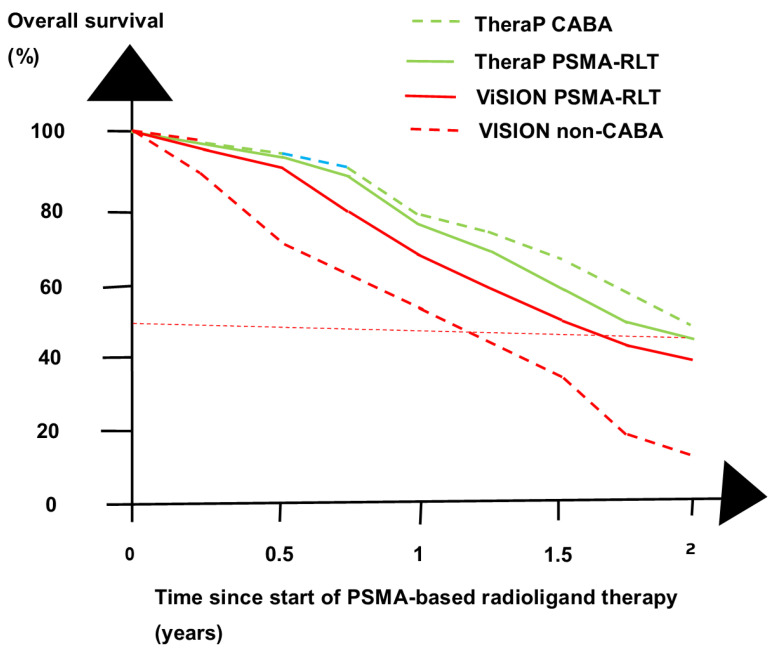
Overall survival (OS) at four time points in the TheraP and the VISION trials. In the VISION trial, the PSMA-RLT group had a significantly better OS than the control group, in contrast to the TheraP trial. The PSMA-RLT groups in the two trials had a grossly similar OS, whereas the CABA-treated TheraP control group (TheraP CABA) had a better OS than the non-cabazitaxel VISION control group (VISION non-CABA).

Progress has been made with PSMA-based theranostics [14,15]. Two RCTs of patients with mCRPC, TheraP and VISION, studied [^177^Lu]Lu-PSMA-617 as a third-line treatment [14,15,16]. ^177^Lu-PSMA-617 increased the rate of patients with PSA decline > 50% relative to that after the control treatments. The ^177^Lu-PSMA-617 groups in the two trials lived grossly similarly, as shown in Figure 2. Many aspects optimize PSMA-RLT for PCa patients [17]. 

[^225^Act]Act-PSMA-RLT is more effective than [^177^Lu]Lu-PSMA-RLT but increases adverse effects such as xerostomia [18,19]. To reduce the adverse effects of ^225^Act-PSMA-RLT, nuclear medicine specialists developed a tandem PSMA-RLT [20]. It has cycles with reduced activity of ^225^Act-PSMA-RLT combined with reduced activity of ^177^Lu-PSMA-RLT. A study compared the tandem with ^177^Lu-PSMA-617 therapy [21]. Tandem gave a significantly higher rate of PSA decline than monotherapy. Another study of the tandem reporting the adverse effects of saliva glands remains a challenge [22].

Our review aims to highlight recent promising RCTs of patients with high-risk PCa. 

## 2. Material and Methods

We searched for relevant RCTs from 2021 to March 2024 in Google Scholar and PubMed. Included were original studies of PSMA-RLT based on small molecules, published in English either as published trials or as presented at major international conferences, irrespective of the phase of PCa and number of patients. Excluded were RCTs where PSMA-RLT was combined with non-established drugs, RCTs that addressed only bone metastases, and RCTs that have not reported outcomes. Effect measures were rate of PSA decline > 50%, radiologic progression-free survival, and overall survival (OS). 

All patients in the RCTs had positive sites on PSMA PET/CT. The RCTs followed the European Association for Nuclear Medicine (EANM) guidelines for PSMA-RLT [24], and classified adverse effects according to the Common Terminology Criteria for Adverse Effects (CTCAE).

A combined *p* value of the individual *p* values of the trials was calculated in STATA by the method of Tobias [25]. 

## 3. Recent RCTs

A trial compared a combination of ABI and PSMA-RLT with PSMA-RLT monotherapy [26]. The combination gave a better outcome than the monotherapy alone. The combination can give a long-lasting complete remission [27]. Table 1 shows recent RCTs. The RCT ENZA-p studied first-line treatment of high-risk patients with mCRPC and compared a combination of ENZA and ^177^Lu-PSMA-617 with ENZA monotherapy [23]. A total of 83 patients had the combination, and 79 patients had the monotherapy. More patients had a PSA decline > 50% after the combination than after the monotherapy: 93% versus 78%, respectively; hazard ratio (HR) = 0.43, *p* < 0.001. 

**Figure 3:**
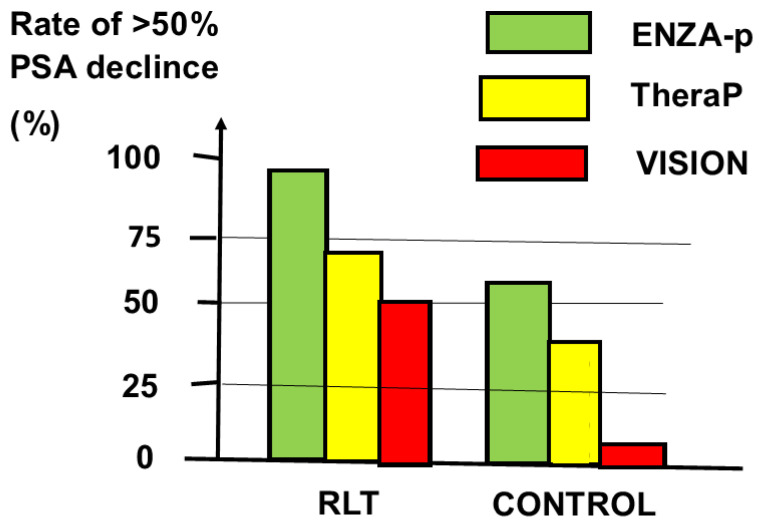
Rate of PSA decline >50% in the ENZA-p, TheraP, and VISION randomized controlled trials [14,15,23]. Patients given PSMA-based radioligand therapy (RLT) had higher rates of PSA decline than control patients given the comparator treatments (CONTROL).

Patients given the combination lived longer free of PSAR than patients given the monotherapy (median 13 months versus 7.8 months, respectively, *p* < 0.0001). The ENZA-p trial had higher rates of PSA decline >50% than the TheraP and VISION trials, as shown in Figure 3. A non-inferiority RCT compared PSMA-RLT and DOC as second-line treatments [28]. 

Other important RCTs have been presented at recent major international conferences. The European Society of Medical Oncology (ESMO) conference in Madrid, Spain, October 2023, presented the PSMAfore trial (NCT04689838) [29]. The trial is an open-label prospective multicenter RCT of second-line treatment of patients with PSMA-PET/CT-positive mCRPC. The control treatment is a second ARPI. Despite the patients having been informed that third-line CABA treatment prolongs life more than a second ARPI, all patients participated in the trial [30].

The included patients had failed on ARPIs, mainly ABI and ENZA. The trial enrolled 468 patients, and compared the outcomes after ^177^Lu-PSMA-617 and after a second ARPI. Patients given ^177^Lu-PSMA-617 lived longer free of radiological progression than patients given a second-line ARPI: median 12 months versus 5.6 months, respectively, HR = 0.41, *p* < 0.0001. The rates of complete radiographic response were 21% and 2.6%, respectively. Most control patients who failed on the second ARPI were later given ^177^Lu-PSMA-617. Grade 3/4 adverse effects were infrequent. 

Another RCT of second-line PSMA-RLT used a related radioligand. The ESMO conference 2022 presented the SPLASH trial (NCT04647526) [31]. It investigated ^177^Lu-PSMA I&T and enrolled 415 patients. The radioligand significantly increased the radiological progression-free survival, as shown in Table 2. 

## 4. Discussion

Our review provides perspectives on the progress in the treatment of PCa. The review focuses on four important RCTs of PSMA-RLT, which investigated 1081 patients. Two studies documented the efficacy of combining ^177^Lu-PSMA-617 with ARPIs [23,26]. As a first-line treatment, the ENZA-p RCT showed a combination of ENZA and ^177^Lu-PSMA-617 increased the effect of ENZA as monotherapy [23], and the Suman study showed that combining ABI and ^177^Lu-PSMA-617 as a second-line treatment increased OS relative to that of ^177^Lu-PSMA-617 monotherapy [26]. The studies favor that nuclear medicine specialists combine PSMA-RLT and ARPI. Another study reported real-world data on ^177^Lu-PSMA-617 as a first-line treatment [32].

The RCTs follow recommendations of the Prostate Cancer Clinical Trials Working Group 3 (PCWG3) [12]. They recommend that drugs (here ^177^Lu-PSMA-617) that are effective as third-line treatments should be studied in earlier phases of PCa. The ENZA-p trial illustrates two promising concepts for PSMA-RLT: early use and use combined with other drugs. The concepts add to those in a review on optimizing PSMA-RLT for PCa patients [17]. 

The TheraP and VISION trials showed that PSMA-RLT as third-line monotherapy is as effective as other established drugs, but has less adverse effects [14,15]. First- and second-line PSMA-RLT improved outcomes, so the efficacy of PSMA-RLT is a class phenomenon. 

The European Association of Nuclear Medicine carried out a Focus 5 conference in Granada, Spain, in 2023 [33]. Leading experts at the conference published their consensus regarding PSMA-RLT for patients with advanced PCa. Thirteen ongoing trials of PSMA-RLT were summarized in another review [34]. The selected trials combined PSMA-RLT with established and non-established drugs. Complementarily, our review gave priority to RCTs that only combined PSMA-RLT with established drugs and summarized the outcomes of RCTs of PSMA-RLT in early phases of PCa. 

**Figure 4:**
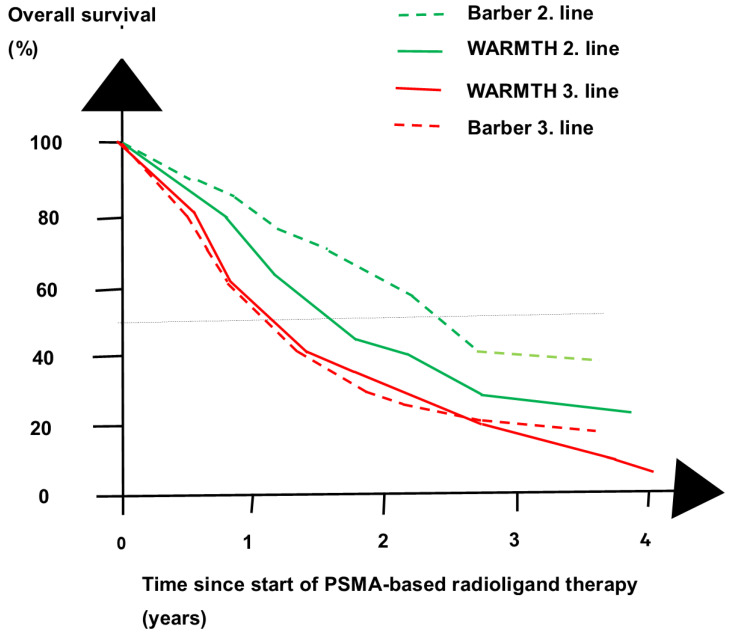
Overall survival (OS) at four time points in two retrospective studies of patients treated with [^177^Lu]Lu-PSMA-617. Patients treated with second-line PSMA-RLT (2. line) lived longer than patients treated with third-line PSMA-RLT (3. line). The figure shows the Barber study [35] with stippled lines and the Ahmadzadehfar (WARMTH) study [36] with full lines. Taxane-naïve patients given PSMA-RLT as second-line treatment shown with green lines lived longer than patients with failure to docetaxel given PSMA-RLT as third-line treatment shown with red lines.

Other studies also investigated the early use of ^177^Lu-PSMA-617. Retrospective studies and a meta-analysis showed that DOC-naïve patients given second-line ^177^Lu-PSMA-617 monotherapy lived longer than DOC-failing patients given third-line ^177^Lu-PSMA-617 monotherapy, as shown in Figure 4 [35,36,37]. The publications included 739 DOC-naïve patients and 1910 DOC-failing patients. DOC-naïve patients lived a median of up to one year longer than DOC-failing patients. A randomized non-inferiority trial compared PSMA-RLT and DOC as second-line treatments [28].

In accordance with the US Federal Drug Administration (FDA) approval of ^177^Lu-PSMA-RLT, the PSMAfore trial included PSMA-RLT as relapse treatment for the control patients after they had failed with the second ARPI. So, the trial did not examine whether ^177^Lu-PSMA-617 had an impact on OS. Sartor et al. defended the trial as giving priority to radiographic progression-free survival [30]. This may be a better study endpoint than the rate of PSA decline > 50%. 

The study design of RCTs is important for the conclusion. The VISION trial concluded that ^177^Lu-PSMA-617 as a third-line treatment significantly improved OS in contrast to the TheraP trial [15,16]. The different conclusions reflect the treatments of the control groups. The TheraP control group was given CABA as a third-line treatment. Previously, the CARD trial showed that third-line treatment with CABA prolonged OS compared with a second ARPI as a third-line treatment [9]. The VISON trial used a “standard of care” treatment of the control group that excluded CABA, Sipuleucel-T (Provenge), and [^223^Ra]Ra dichloride (Xofigo). As expected, the TheraP control group lived longer than the VISION control, as shown in Figure 2. 

Subgroups of patients have an especially high effect from ^177^Lu-PSMA-617. Retrospective studies have shown that oligometastatic mCRPC patients treated with PSMA-RLT lived impressively long [38]. Further, retrospective studies showed that patients treated with PSMA-RLT for lymph node metastases (LNM) lived longer than patients with bone, lung, and liver metastases [39,40]. Docetaxel (DOC)-naïve LNM patients lived longer free of PSAR than LNM patients who had failed with DOC [41].

It is a challenge for PSMA-RLT that some PSMA PET-positive patients do not respond [42]. Resistance to PSMA-RLT may be due to molecular biology [43]. A study indicated that androgen receptor gene amplification contributes to the resistance [44]. Similarly, some patients are resistant to radiation therapy. Studies have shown molecular mechanisms for radiotherapy resistance [45], but it remains to be shown whether the mechanisms for resistance to PSMA-RLT are similar to those for resistance to radiation therapy. 

Recent progress in the management of patients with mCRPC has implications. The terms “first-line, second-line, and third-line treatment” reflect a routine sequence of monotherapies. Only half of the patients with mCRPC who failed with the first-line treatment underwent a second-line treatment, and only half of the patients who failed with the second-line treatment underwent a third-line treatment [8,10]. Now patients with mCRPC are increasingly treated with doublets or triplets, so future trials should categorize patients by previous treatments with ARPIs and taxanes, and not by the “line of treatment”.

Increasingly, patients undergo staging with PSMA PET/CT. Where units have access to PSMA PET/CT, more than half of the patients undergo initial staging with PSMA PET/CT, and nearly all patients with PSAR undergo restaging with PSMA PET/CT. This motivates a shift from a TNM staging based on conventional imaging (cT, cN, cM) reflecting positive findings in the prostate bed, lymph nodes, and distant organs to a staging based on PSMA PET/CT as miT, miN, and miM [46]. The stage based on PSMA PET/CT is closer to the pathologic stage (pT, pN, pM), the gold standard reference, than the stage based on conventional imaging.

**Figure 5:**
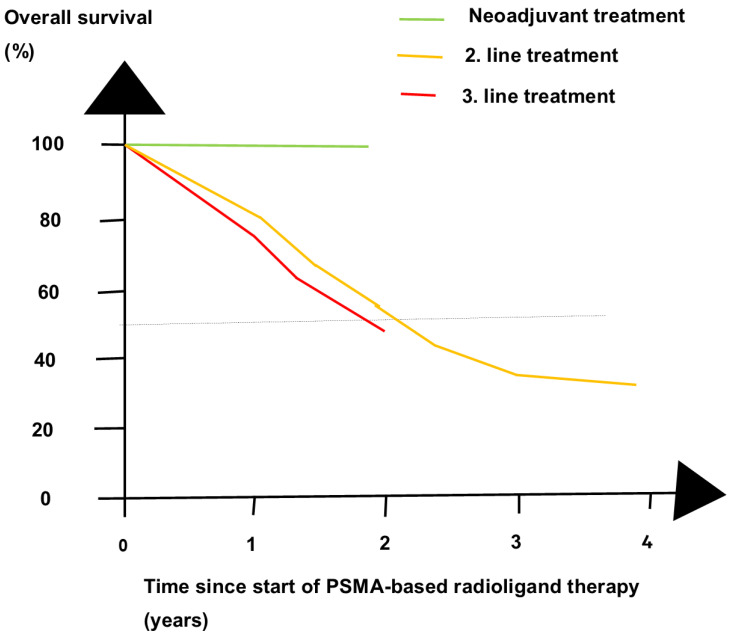
Overall survival (OS) at four time points for radioligand therapy in three phases of prostate cancer. PSMA-RLT as neoadjuvant treatment of patients before radical prostatectomy gave a longer OS than PSMA-RLT as second-line (2. line) and third-line (3. line) treatment of patients with metastatic castration-resistant prostate cancer.

Neoadjuvant treatment has a growing role in oncology. However, neoadjuvant treatment of patients with high-risk PCa has not yet been established. Whereas neoadjuvant ADT was ineffective [47,48], ENZA and darolutamide are more effective. DOC may add to the effect of ADT on radiation therapy [48]. A meta-analysis summarized neoadjuvant treatments in PCa [49]. Two recent pilot studies indicated that ^177^Lu-PSMA-617 is effective as a neoadjuvant treatment [50,51]. It had a brilliant impact on OS, as demonstrated in Figure 5. Similarly, neoadjuvant treatment is effective for patients with breast cancer [52], non-small-cell lung cancer [53], muscle-invasive urinary bladder cancer [54], and rectal cancer [55]. 

[^161^Tb]Tb is another radioisotope with potential for PSMA-RLT of PCa [56]. Mice models of PCa showed [^161^Tb]Tb-PSMA-RLT was more effective than [^177^Lu]Lu-PSMA-RLT. In two recent case reports of patients with mCRPC, [^161^Tb]Tb-PSMA-RLT gave promising results [57,58]. New trials further investigate the dosimetry and efficacy of [^161^Tb]Tb-PSMA-RLT in patients with PCa [59,60].

Our review has strengths and limitations. As a strength, RCTs gave consistent positive findings. As a limitation, the FDA approved ^177^Lu-PSMA-167 only as third-line monotherapy of patients with mCRPC. Two RCTs are published only as conference abstracts. Patients with *BRAC2* mutations can be treated with a PARP inhibitor, such as olaparib. An RCT, LuPARP, investigates a combination of PSMA-RLT and olaparib [61].

## 5. Future Directions 

The ENZA-p trial raises questions: would neoadjuvant PSMA-RLT of high-risk PCa patients be more effective than first-line treatment of patients with mCRPC? Would a combination of ADT, ENZA, and PSMA-RLT for patients with mHSPC be more effective than a combination of ENZA and PSMA-RLT for patients with mCRPC? Trials that address the questions are being developed.

## 6. Conclusions

Patients with PCa may gain from PSMA-RLT used in early phases of the cancer, and used in combination with other established drugs, especially ARPIs. 

## Figures and Tables

**Table 1:** RCTs investigating early PSMA-RLT in patients with mCRPC.

Phase of Treatment	Author/NCT Number	Reference	Name of Trial	Type of RLT	No of Patients
First-line treatment	Emmett 2024	[23]	ENZA-p	PSMA-617	162
Second-line treatment	Satapathy 2023	[28]	NR		40
	NCT04689838	[29,30]	PSMAfore	PSMA-617	467
	Hansen 2022	[31]	SPLASH	PSMA I&T	412
Overall total					1081

The table counts the enrolled or planned enrolled number of patients in the RCTs of PSMA-RLT. For one RCT, the table refers to the trial number registered at ClinicalTrials.gov. Abbreviation: NR = not reported.

**Table 2:** Radiological progression-free survival gain with PSMA-RLT in RCTs.

Trial	Reference	Radiological Progression-Free Survival (Months)	Hazard Ratio	Single *p* Value	Combined *p* Value
					
					
					
PSMAfore	[29]	12 vs. 5.6	0.41	<0.0001	
SPLASH	[31]	9.5 vs. 6	0.71	0.0088	
Combined					<6.9 × 10^−6^

## List of Abbreviations

ABI	abiraterone
Act	Actinium
ADT	androgen deprivation therapy
ARPI	androgen receptor pathway inhibitor
BCR	Biochemical recurrence
CABA	cabazitaxel
CT	computed tomography
DOC	docetaxel
ENZA	enzalutamide
ENZA-P	randomized controlled phase 2 trial of the combination of enzalutamide and PSMA-based radioligand therapy
ESMO	European Society of Medical Oncology
LNM	lymph node metastases
Lu	lutetium
mCRPC	metastatic castration-resistant prostate cancer
mHSPC	metastatic hormone-sensitive prostate cancer
nmCRPC	nonmetastatic castration-resistant prostate cancer
OS	overall survival
PCa	prostate cancer
PCWG3	Prostate Cancer Clinical Trials Working Group 3
PET	positron emission tomography
PSA	prostate-specific antigen
PSAR	prostate-specific antigen relapse
PSMA	prostate-specific membrane antigen
Ra	Radium
RCT	randomized controlled trial
RLT	radioligand therapy
rPFS	radiological progression-free survival
Tb	Terbium
